# Controlled Heat and Humidity-Based Treatment for the Reuse of Personal Protective Equipment: A Pragmatic Proof-of-Concept to Address the Mass Shortage of Surgical Masks and N95/FFP2 Respirators and to Prevent the SARS-CoV2 Transmission

**DOI:** 10.3389/fmed.2020.584036

**Published:** 2020-10-20

**Authors:** Louis Bernard, Guillaume Desoubeaux, Elsa Bodier-Montagutelli, Jeoffrey Pardessus, Déborah Brea, Laurine Allimonnier, Sébastien Eymieux, Pierre-Ivan Raynal, Virginie Vasseur, Laurent Vecellio, Ludovic Mathé, Antoine Guillon, Philippe Lanotte, Jérémie Pourchez, Paul O. Verhoeven, Stéphane Esnouf, Muriel Ferry, Nicolas Eterradossi, Yannick Blanchard, Paul Brown, Philippe Roingeard, Jean-Pierre Alcaraz, Philippe Cinquin, Mustapha Si-Tahar, Nathalie Heuzé-Vourc'h

**Affiliations:** ^1^Médecine interne et maladies infectieuses, CHU de Tours, Tours, France; ^2^Université de Tours, Tours, France; ^3^Parasitologie–mycologie-médecine tropicale, CHU de Tours, Tours, France; ^4^Inserm U1100, Centre d'étude des pathologies respiratoires (CEPR), Tours, France; ^5^Pharmacie à usage intérieure, CHU de Tours, Tours, France; ^6^Biologie cellulaire–Microscopie électronique, CHU de Tours, Tours, France; ^7^UMR Inserm U1259-Morphogénèse et antigénicité du VIH et des virus des hépatites, Tours, France; ^8^Blanchisserie centrale GCS NOT, CHU de Tours, Tours, France; ^9^Médecine intensive–Réanimation, CHU de Tours et Université de Tours, Tours, France; ^10^Bactériologie–Virologie–Hygiène hospitalière, CHU de Tours, Tours, France; ^11^ISP Equipe 5-Bactéries et Risque Materno-fœtale, INRAE, Nouzilly, France; ^12^Mines Saint-Etienne, Université Jean Monnet, INSERM, U 1059 Sainbiose, Centre CIS, Saint-Etienne, France; ^13^GIMAP, EA 3064, Université Jean Monnet, Université de Lyon, Saint-Etienne, France; ^14^Service des Agents Infectieux et d'Hygiène, CHU de St-Etienne, Saint-Etienne, France; ^15^Service d'Étude du Comportement des Radionucléides (SECR), CEA, Université Paris Saclay, Gif-sur-Yvette, France; ^16^French Agency for Food Environmental and Occupational Health Safety (Anses), Ploufragan, France; ^17^TIMC-IMAG, UMR5525 Univ. Grenoble Alpes-CNRS, La Tronche, France; ^18^CIC-IT1406 INSERM/CHU Grenoble Alpes/Univ. Grenoble Alpes, La Tronche, France

**Keywords:** SARS–CoV-2, facemask, recyclibility, surgical face masks, COVID-19, heating, FFP2/N95, coronavirus

## Abstract

**Background:** The coronavirus infectious disease-2019 (COVID-19) pandemic has led to an unprecedented shortage of healthcare resources, primarily personal protective equipment like surgical masks, and N95/filtering face piece type 2 (FFP2) respirators.

**Objective:** Reuse of surgical masks and N95/FFP2 respirators may circumvent the supply chain constraints and thus overcome mass shortage. Methods, design, setting, and measurement: Herein, we tested the effects of dry- and moist-air controlled heating treatment on structure and chemical integrity, decontamination yield, and filtration performance of surgical masks and FFP2 respirators.

**Results:** We found that treatment in a climate chamber at 70°C during 1 h with 75% humidity rate was adequate for enabling substantial decontamination of both respiratory viruses, oropharyngeal bacteria, and model animal coronaviuses, while maintaining a satisfying filtering capacity.

**Limitations:** Further studies are now required to confirm the feasibility of the whole process during routine practice.

**Conclusion:** Our findings provide compelling evidence for the recycling of pre-used surgical masks and N95/FFP2 respirators in case of imminent mass shortfall.

## Highlights

- A worldwide mass shortage of surgical masks and N95/FFP2 respirators has been observed during the coronavirus infectious disease-2019 (COVID-19) pandemic;
- Alternative means for recycling pre-used face masks are warranted in such a context of sanitary crisis;
- A moist heating treatment results in a satisfactory decontamination of critical respiratory pathogens while preserving the structural integrity and filtration efficiency of protective masks.

## Introduction

Severe acute respiratory syndrome coronavirus 2 (also referred to as SARS-CoV-2) is responsible for the coronavirus infectious disease-2019 (COVID-19) pandemic. SARS-CoV-2 remains viable over several hours on different inert surfaces and up to 3 h in the air (1). During previous other epidemics, the airborne route of transmission was already associated with nosocomial super-spreading events (2). Although not fully elucidated so far, transmission of SARS-CoV-2 may occur partly by aerosol droplets and contaminated postilions of aerodynamic diameter ranging from 0.25 to 3.0 μm (3). Accordingly, any face-to-face contact closer than ≤6 feet to a symptomatic patient should be considered significant exposure, if sustained for at least a few minutes (4).

In order to reduce the risk of interindividual contamination (5), most governments, medical societies, and health associations agreed to recommend to healthcare workers and caregivers systematic wearing of protective face masks, like surgical masks and N95/filtering face piece (FFP) respirators to cover the mouth and nose during the COVID-19 pandemic (to be complemented by meticulous hand hygiene, eye protection, gloves, and gown wearing). Approved protective masks are composed of several layers of nanofibers made with polypropylene (6). Surgical masks are primarily designed to prevent transmission of pathogens from infected patients wearing them to others and from contaminating their surroundings and direct environment (5). Additionally, FFP respirators protect noninfected healthy people wearing them from inhalation of aerosol particles. According to European standards, FFPs are sorted in three distinct subclasses depending on their aerosol filtration efficiency and leakage percentages. During the COVID-19 pandemic, caregivers are encouraged to wear at least FFP2-grade respirators (specifications close to N95 respirators in the USA), coming in contact with patients infected with SARS-CoV-2 or suspected of being so. Both surgical masks and N95/FFP2 respirators are single-use disposable devices, and most industrial manufacturers are currently overwhelmed by massive orders. Thus, several countries and health facilities are now suffering from in- and out-hospital mass shortage of protective surgical masks and N95/FFP2 respirators.

Therefore, in the present context of world sanitary emergency, alternative processes allowing to extend the existing on-hand supplies are critically required to offer satisfying respiratory protective means for all healthcare workers (7). Production of fabric masks (e.g., with non-cellulose synthetic fibers based on nonwoven polypropylene [Spunbond, Meltblown, Spunbond (SMS)]) (5) or application of different decontamination means to pre-used protective masks has been urgently assessed. Many strategies are unsatisfactory (8, 9), insufficiently documented, or leading to poor decontamination yields and loss of filtration performances. Moreover, both the practical transposition of all these treatment procedures to each hospital service, attendant care, and nursing department and the wearers' comfort have often been neglected. Thanks to an incredible international multidisciplinary effort, promising disinfecting processes are emerging and some are already in application in “real-life conditions.”

Herein, we demonstrate the benefits of an easy-to-use recycling solution that will reduce the overall burden on mass shortage in healthcare facilities using a heating stage at 70°C, a temperature known to inactivate the SARS-CoV-2 (10). Hence, we provide a simple procedure that efficiently decontaminates surgical masks and FFP2 respirators from respiratory pathogens while preserving filtration performances.

## Materials and Methods

### Protective Masks

All assays on oropharyngeal bacteria, influenza virus, and filtration performances were carried out using conventional elastic surgical masks (different brands including THF type II R 3 Plis®, CA Diffusion, Halluin, France) and FFP type 2 (FFP2) respirators (RP2_M®, CA Diffusion). Inactivation assays on surrogate animal coronaviruses were performed using surgical (THF type IIR CA1960®, CA Diffusion) and FFP2 (FFP2 NRD type IIR 2192S-WH®, Medicom, Saint Barthélémy d'Anjou, France). All were certified by EN 149:2001+A1:2009 NF or EN 14683+AC standards.

### Model Strains of Oropharyngeal Bacteria

*Streptococcus pyogenes* (isolate 19-103100), *Staphylococcus aureus* (strains ATCC 29213 and ATCC 6538), and *Haemophilus influenzae* strains (isolate CIP776) were grown in heart–brain liquid medium (BD Brain Heart infusion broth®, Beckton Dickinson, Rungis, France) or in Muller–Hinton® agar (bioMérieux, Craponne, France).

For titration, bacteria were diluted 10-fold up to 1:10,000, and 50 μl of each dilution were then deposited onto agar plates, trypticase soy agar TSH® (bioMérieux) for *S. aureus* and *S. pyogenes* or onto Chocolate agar PolyViteX® agar (bioMérieux) for *H. influenzae*, before incubation at 37°C and subsequent counting of the number of colony-forming units (CFU/ml).

### Model Strains of Respiratory Viruses

The influenza A H3N2/Scotland/20/74 strain was prepared as previously described (11) and cultured onto canine kidney epithelial mycoplasma-free Madin-Darby canine kidney (MDCK) cells (ATCC CCL-34) with minimum essential medium-Eagle (MEM) supplemented with 10% fetal bovine serum (FBS) and 1% penicillin/streptomycin.

Two animal coronaviruses were used as surrogates for SARS-CoV-2. Porcine epidemic diarrhea virus (PEDV) strain CV777 (12) was grown on Vero cells (ATCC® CCL-81) in MEM (Thermo Fisher Scientific, Waltham, MA, USA) supplemented with 0.3% tryptone phosphate broth, 0.02% yeast extract (12) (adjuvants to culture media cells), 1% penicillin/streptomycin, and 10 μg/ml trypsin (13). Infectious bronchitis virus of chicken (IBV) strain Mass 41 (14) was propagated on primary cultures of kidney cells prepared from specific pathogen-free chicken embryos of 19 days of age (14), maintained in BHK-21 medium (Gibco, Cergy-Pontoise, France), supplemented with 0.15% tryptone phosphate broth, 1% penicillin/streptomycin, 1.5% of FBS, and the pH was adjusted to 7.2 with NaHCO_3_ (15). Suspensions of IBV and PEDV were prepared in culture medium containing 20% FBS before inoculation onto masks.

Influenza virus titration was performed using the plaque-forming unit (PFU) method adapted from Matrosovich et al. (16). Briefly, six-well cell culture plates were seeded at 1.0 × 10^6^ MDCK cells/well. One day later, cells were washed with MEM buffer and infected at 37°C with 400 μl of serial dilutions of the sample. Plates were gently shaken every 10 min for 1 h. Then, each well was covered with 3 ml of a mixture of MEM buffer, 1.2% Avicel® (Dupont, Copenhagen, Denmark), 1% penicillin/streptomycin, and 1 μg/ml of TPCK-Trypsin® (Thermo Fisher Scientific). Plates were further incubated at 37°C for 72 h. After two washings in PBS buffer, cell layers were stained with a solution containing 10% crystal violet oxalate, 10% formaldehyde, and 20% ethanol, and plaques were counted. Viral titers were finally expressed as PFU/ml. Animal PEDV and IBV coronaviruses were titrated according to Reed and Muench (17), and virus titers were expressed as TCID_50_/ml (50% tissue culture infective dose per milliliter), as calculated based on immunoperoxidase monolayer assay on Vero cells (PEDV) or immunofluorescent assay on primary chicken kidney cells (IBV) (18), using pathogen-specific pig and chicken anti-sera.

### Dry- and Moist-Air Heating Treatment Procedures

The surgical masks and FFP2 respirators were inserted into ISO11607-certified sterilization bags (NF EN 868-5, Amcor, Zürich, Switzerland) and submitted to different heat treatments based on either single usual runs (70°C−15 min, 70°C−60 min, 90°C−3 h, 100°C for 60 min, or 120°C−10 min) in the tumble-drying machine (Kannegiesser®, Nanterre, France) with rotation but without detergent or single transit [70°C−1 h no humidity or 70°C−1 h with 75% humidity rate (HR)] in an HPP260® constant climate chamber (Memmert GmbH, Schwabach, Germany). For the coronavirus experiments, the experimentally contaminated protective masks were inserted into a Binder KBF115® climate chamber (Binder GmbH, Tuttlingen, Germany) and submitted to a single heat treatment of 70°C−1 h–with 75% HR.

### Assessment of the Structural and Chemical Integrity

After the heating treatment, surgical masks and FFP2 respirators underwent successive evaluations that were carried out as GO/NO GO steps in order to ensure the preservation of their integrity and their function (Supplementary Figure 1). For GO/NO GO Step 1, unused surgical masks, and FFP2 respirators were thoroughly observed for obvious changes in physical appearance (color, shape, and size), and their ultrastructure was compared to untreated masks using scanning electron microscopy [(SEM) Ultra Plus® FEG, Zeiss, Oberkochen, Germany]. For such a purpose, mask layers were first coated with 40-Å platinum using a PECS 682® apparatus (Gatan, Pleasanton, CA, USA).

At the molecular level, modifications of the treated (unused) surgical masks and FFP2 respirators were evaluated by Fourier-transform infrared spectroscopy equipped with single-reflection diamond attenuated total reflectance (ATR) accessory (i.e., Vertex 70v FT-IR® spectrometer, Bruker, Billerica, MA, USA) with a Golden Gate® (Specac, Orpington, United Kingdom): acquisition was recorded between 4,000 and 600 cm^−1^, with a resolution of 4 cm^−1^ and 64 scan repetitions. Volatile molecules trapped in the film were identified by thermal desorption (TD) through a TD 350® thermo-desorber (PerkinElmer, Courtaboeuf, France) coupled to a GC-6890® gas chromatography (GC) associated with a MS5973N® mass spectrometer [(MS) Agilent, Les Ulis, France] under TD conditions at 140°C and under helium for 10 min (19). Only the treatment conditions that allowed correct preservation of the masks integrity were kept for further assessments described below.

### Assessment of the Decontamination Yield

For GO/NO GO Step 2 (Supplementary Figure 1), 50 μl of each pathogen suspension were deposited onto a delimited area of protective surgical masks and FFP2 respirators on either the inner or the outer lining. Then, the masks were incubated at 37°C for 1 h to dry the pathogen suspension and further submitted to dry-air or moist-air heating treatments, as described above. Control masks (with deposition of bacteria or virus suspension, but no heating treatment) were stored at 4–6°C for the same duration. Thereafter, all the afore-delimited areas were cut out, placed into 2-ml sterile water or culture medium (when loaded with either bacteria or viruses, respectively, in order to resuspend the residual pathogens), and then mixed. Next, the suspensions were diluted and analyzed as described above for titration. Since the relative extraction rates from the FFP2 respirator fibers were estimated at 22 and 15% for bacteria (*S. aureus*) and virus (influenza A H3N2/Scotland/20/74), respectively, the lower limit of detection (LLOD) was thus established at <100 CFU/ml for bacteria and <17 PFU/ml for influenza virus. The extraction rate was 0.06 and 10% for the IBV and PEDV (a 10-fold dilution was necessary to dilute the residual FBS, which interfered with viral isolation). The extraction rates (ratio between infectious titers of initial viral inoculum and virus eluted from masks) were 16.6 and 3% for the PEDV and IBV, respectively. The LLOD for the PEDV and IBV re-isolation procedures was determined by 10-fold serial dilutions and was 10^1.5^ TCID_50_/ml for both viruses. The TCID_50_ reduction after moist-air heating treatment was calculated to be at least the difference between the infectious titer of eluted virus and the LLOD. Only the treatment condition that allowed correct achievement of decontamination yield were kept for further assessments described below.

### Assessment of the Bacterial Filtration

For GO/NO GO Step 3, the evaluation of the filtration efficacy of the surgical masks was performed following the EN 14683:2019 standard (20) (Supplementary Figure 1). Briefly, a specimen of the inside surgical mask material was clamped between a six-stage viable Andersen cascade impactor and an aerosol chamber (glass, 445 mm long and 60 mm in external diameter; Supplementary Figure 2, left panel). Aerosolization of 3.0 ± 0.3 μm droplets from a 3-ml suspension of *S. aureus* (ATCC 6538) was achieved by the E-Flow® mesh nebulizer (Pari GmbH, Starnberg, Germany) to maintain a bacterial challenge (2,200 ± 500 CFU per test) during a 1-min nebulization. Each test specimen was conditioned at 21 ± 5°C and 85 ± 5% HR for the time required to bring them into equilibrium with atmosphere prior to testing. Finally, the filtration efficiency of the masks was expressed as a percentage of the CFU initially present in the challenge aerosol that passed through the material.

### Assessment of the Viral Filtration

For the rest of GO/NO GO Step 3 (Supplementary Figure 1), aerosolization of a suspension at 3.5 × 10^7^ PFU/ml influenza A H3N2/Scotland/20/74 virus strain was achieved by the Aerogen Solo® mesh nebulizer (Aerogen, Galway, Ireland) (21) and using the experimental setup described in Supplementary Figure 2 (right panel). The size of the droplets (3.6 ± 0.1 μm) was similar to the EN 14683:2019 standard that is dedicated to the assessment of medical devices for usage against respiratory pathogens. After nebulization, the virus was collected in a BioSampler® device (SKC, Eighty Four, PA, USA) filled with 5 ml of MEM buffer, and the percentage of viral particles passing through the masks was determined by virus titration as aforementioned. Considering the experimental setting, the LLOD for filtration was estimated at 2.5 PFU/ml.

### Measurement of the Inspiratory Resistance

For GO/NO GO Step 3 bis., an original mounting using a 4000 series® digital flow meter (TSI, Marseille, France) was exploited for measuring the inspiratory resistance of the surgical masks and FFP2 respirators before and after treatments (Supplementary Figure 3). Two pressure gauges (Magnehelic 2000-0®, Dwyer Instruments, Suresnes, France) continuously controlled the pressure differential between the flow meter and the pump. Different inspiratory flow rates were tested from 15 to 60 L/min. The difference in inspiratory resistance of heat-treated masks compared to control masks was expressed in Pascal (Pa) per square centimeter.

## Results

**Figure 1:**
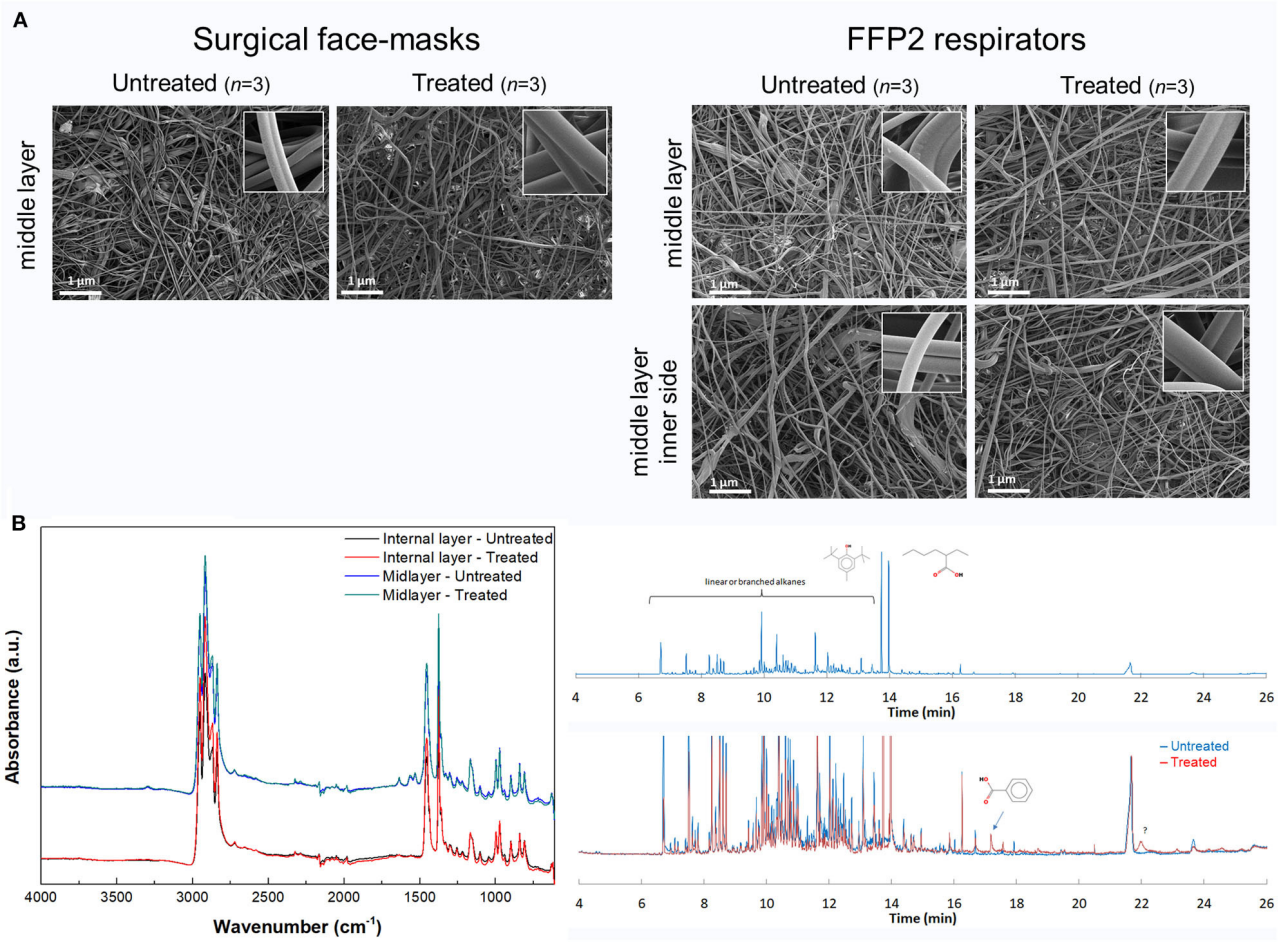
**(A)** Observation through scanning electron microscopy of the middle layer of surgical masks and filtering face piece type 2 (FFP2) respirators whether untreated or treated by moist-air heating at 70°C (75% humidity rate (HR) during 1 h) in the climate chamber. The inner panels show the correct structural integrity of polypropylene nanofibers at higher magnification. **(B)** Assessment of the molecular modifications of FFP2 respirators treated by moist-air heating at 70°C (75% HR during 1 h) in the climate chamber compared to untreated FFP2, as observed through Fourier-transform infrared attenduated total reflection (FTIR-ATR) (left panel) and thermal desorption–gas chromatography–mass spectrometry (TD-GC-MS) (right panel). The FFP2 respirator layers were confirmed as composed of polypropylene only, except for the mid-layer of treated/untreated FFP2 respirators that also presented four additional bands centered at 3,295, 1,640, 1,565, and 1,530 cm^−1^, which probably correspond to a molecule of the amide family; this hypothesis is supported by the fact that this kind of molecule is known to be an effective process agent during the melt-blown process of the polypropylene fibers (22). The left panel shows only the internal and mid-layer as examples. The chromatogram displayed in the upper right panel showed the four layers of the untreated FFP2 respirators altogether. It indicated a low quantity of molecules: most of them were linear and branched alkanes. The two main peaks at 14 min represented butylated hydroxytoluene (BHT), i.e., a very well-known antioxidant, and 2-ethylhexanoic acid. In the lower right panel is superimposed the chromatogram of the treated FFP2 respirators to the untreated ones. Only two very weak new peaks were observed: the first one corresponds to benzoic acid, the second one being unidentified. a.u., arbitrary units; cm^−1^, per centimeter; min, minute.

The global design of the study is summarized in Supplementary Figure 1. Following the runs in the tumble-drying machine, only the treatment condition at 70°C during 15 or 60 min allowed complete preservation of the global structure and the integrity of the surgical masks and FFP2 respirator nanofibers. In contrast, longer or hotter processes were deleterious for the quality, with slight or rough obvious destruction. After one 60-min cycle in the climate chamber at 70°C and 75% HR, no macro- or micro-alterations were observed (Figure 1A). Contrary to the autoclaving process, the surgical masks and FFP2 respirators were not wet in such a condition (GO/NO GO Step 1, positively checked). The same finding was observed after 3–5 iterative cycles of moist heating (data not shown). After moist-air heating treatment, there was no proof of (oxidization-based or chemical) alteration at the molecular level (Figure 1B) of any layer of the surgical masks and FFP2 respirators. This was also the case for the elastic rubber band (not shown). Besides, the mask layers were confirmed as composed of polypropylene only, except for the mid-layer of treated/untreated surgical masks and FFP2 respirators that also presented four additional bands centered at 3,300, 1,642, 1,564, and 723 cm^−1^ and 3,295, 1,640, 1,565, and 1,530 cm^−1^, respectively, which probably correspond to a molecule of the amide family. TD-GC-MS chromatogram indicated that two new molecules were trapped in the treated FFP2 respirators (in comparison with untreated respirators): they actually corresponded to benzoic acid and an unidentified compound (Figure 1B). Nevertheless, their respective signal intensities were so weak that no one could conclude to significant modification of any of the four layers of the FFP2 respirators. The same comment applied to the elastic rubber bands (not shown). Altogether, only the treatment conditions with dry-air heating at 70°C for 15–60 min and moist-air heating at 70°C for 1 h were thus kept for further investigations (Supplementary Figure 1).

**Figure 2:**
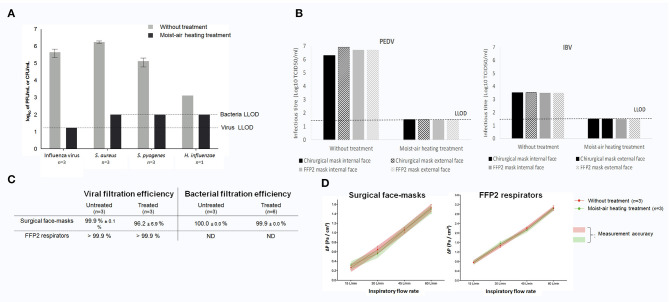
**(A)** Decontamination yields obtained after dry- or moist-air heating treatment for influenza A H3N2/Scotland/20/74 virus and oropharyngeal bacteria. **(B)** Decontamination yields obtained after moist-air heating treatment for swine porcine epidemic diarrhea virus [(PEDV) left panel] and avian infectious bronchitis virus of chicken [(IBV) right panel] coronaviruses. Lack of virus re-isolation was arbitrarily indicated in the figure as a titer equal to the lower limit of detection (LLOD) of the re-isolation process. **(C)** Filtration properties of surgical masks (upper panel) and filtering face piece type 2 (FFP2) respirators (lower panel) for the influenza A H3N2/Scotland/20/74 virus strain and for *Staphylococcus aureus* ATCC 6538 (on surgical masks only for the latter as specified by EN 14683 guidelines). **(D)** Measurement of inspiratory resistance for surgical masks (left panel) and FFP2 respirators (right panel). All results are expressed as mean ± SEM. CFU, colony-forming unit; ΔP, differential of pressure; L/min, liter per minute; ND, not determined; Pa/cm^2^, Pascal per square centimeter; PFU, particle-forming unit; SEM, standard error of the mean.

Decontamination assay showed the inability of dry-air heating (70°C, 15 or 60 min) to drastically reduce the number of bacteria and the virus titers (by 0 to −1.0 log_10_ only; data not shown). In contrast, moist-air heating in climate chamber (70°C, 75% HR for 60 min) resulted in a remarkable decrease of oropharyngeal pathogens and influenza virus (GO/NO GO Step 2, positively checked): −6.0 log_10_ for *S. aureus*, −5.0 log_10_ for *S. pyogenes*, −3.0 log_10_ for *H. influenza*, and −5.5 log_10_ for the influenza A H3N2/Scotland/20/74 virus strain (*P* < 0.05) (Figure 2A), which corresponds to the maximum based on the limit of detection of each assay. The moist heating treatment also allowed inactivation of the two surrogate animal coronaviruses, as evidenced by TCID_50_ reductions of at least −5 log_10_ and −2 log_10_ for PEDV or IBV, respectively, irrespective of the kind of protective masks and mask side (inside/outside linings) tested (Figure 2B). Therefore, only the moist-air treatment condition (70°C for 60 min with 75% HR) was kept for further analyses.

After treatment in the climate chamber at 70°C during 60 min with 75% HR, the bacterial filtration efficiency of surgical masks for *S. aureus* was assessed at 100.0 ± 0.0% for the moist-heated treatment (vs. 99.9 ± 0.0% for untreated masks; right panel of Figure 2C, GO/NO GO Step 3, positively checked), following the EN 14683 standard. The viral filtration performances for the influenza virus were 96.2 ± 6.9% with the surgical masks and >99.9% with the FFP2 respirators (left panel of Figure 2C, GO/NO GO Step 3, positively checked). Noteworthy, filtration of fluorescein, using the same experimental setup, resulted in 98.9 and 99.9% filtration for surgical masks (*n* = 2) and FFP2 respirators (*n* = 2), respectively (data not shown). The discrepancy between the virus and fluorescein filtration rate for surgical masks was due to the heterogeneous and low viability of the influenza A H3N2/Scotland/20/74 virus during nebulization/collection (data not shown).

Whatever the inspiratory flow, no difference were observed regarding the resistance parameters of control masks and treated ones for both surgical masks and FFP2 respirators (Figure 2D, GO/NO GO Step 3 bis., positively checked).

## Discussion

SARS-CoV-2 is a virus with an outer envelope, which means that it is theoretically very sensitive to conventional decontamination methods. Few years ago during the H1N1 influenza pandemic, the National Academy of Medicine (formerly known as Institute of Medicine, USA) already suggested that simple decontamination techniques [e.g., bleach, ethylene oxide, ultraviolet (*UV*) germicidal irradiation, hydrogen peroxide gas, microwave oven irradiation, etc. (8, 23)] should be deeply investigated in an effort to extend the service life of protective masks (24). Unfortunately, many processes showed that either the structural integrity or the filtration performance of such treated mask could be drastically altered following multiple exposure to aerosols, chemicals, and extreme temperature (25). Furthermore, inappropriate decontamination may be a potential risk of infection since recycled surgical masks and N95/FFP2 respirators may become a reservoir for pathogens (26). For instance, UV treatment was found to destroy the outer polypropylene nanofibers. Likewise, dry heating ≥160°C, as well as 70% isopropyl alcohol spraying, caused significant filter degradation (8). Gamma irradiation with 20 kGy (2 MRad) was demonstrated as sufficient for inactivating the viruses, but studies showed possible deformation of the masks, compromising the inner filtering layer of N95/FFP2 respirators and also the correct mask fitting on the face (9). However, in light of the current sanitary emergency, the Atlanta Centers for Disease Control and Prevention (CDC) recently provided guidelines for “crisis alternate strategies,” including the use of improvised homemade or treated masks (27). Thanks to several national task forces, comprising both academic laboratories and private companies, evaluating the reuse of surgical masks and N95/FFP2 respirators, promising decontamination processes have emerged during the COVID-19 sanitary crisis. One of the most advanced is hydrogen peroxide vapor, already applied in a clinical setting in the USA, and showing decontamination and maintenance of post-decontamination performances on N95 respirators (28). Hopefully, wearers will not suffer discomfort due to residual hydrogen peroxide odor, as previously described (29).

Tumble-drying machines are cosmopolitan equipment, which is commonly used at home or in the hospital for laundry. They offer the possibility to heat at 70°C—a temperature reported to *in vitro* inactivate the SARS-CoV-2 by decreasing its TCID_50_ titer in Vero-E6 cells from 6.8 log_10_ to undetectable level after 5 min of exposure—or higher temperatures (10). In the past, such hot-air drying processes already showed good decontamination performance for reducing the bacteria and virus burden (30, 31), but only at ≥92°C (32). Unfortunately, in the present study, we evidenced that dry-air heating in the tumble machine failed to reach satisfying decontamination yields and even generated sometimes degradation of the material with exposure to moderate to extreme hot temperatures >70°C and/or the mechanical frictions inside the machine. In contrast, and as suggested by preliminary studies (33, 34), we showed herein that a unique step of moist-air heating at 70°C during 1 h in climate chamber, with 75% HR, was efficient on relevant surrogate viruses of SARS-CoV-2. Overall, our findings showed that this treatment did not generate an alteration of the surgical masks and FFP2 respirators at the structural and molecular levels, while it ensured effective bacterial/viral decontamination and allowed conservation of their filtration efficiency and resistance performances. Interestingly, the masks were not wet after climate chamber decontamination (contrary to autoclaving), avoiding an additional drying step that may be deleterious.

To date, climate chambers are mostly reserved to hospital pharmacies or pharmaceutical companies. They enable traceable control of the temperatures, and they can provide 3–80% HR inside. One could easily imagine the feasibility of implementing and defining a logical circuit for recycling the protective surgical masks and N95/FFP2 respirators and then ensuring their return to their first user—a critical item to be considered to ensure acceptability of reuse.

Through the aforementioned method, one can say that we have totally fulfilled the conditions recommended by The Center for Infectious Disease Research and Policy of Minnesota University, which states that, in the case of a pandemic, an acceptable decontamination method must render the organism (or a closely related surrogate) nonviable and not diminish filter and fit performance, respirator integrity and structure, or comfort, odor, and wears. Moreover, the moist heating treatment has also been recently included in pandemic crisis standards of care decontamination recommendations by the Atlanta CDC based on previous studies showing efficient decontamination of closely related virus surrogates (H1N1 virus) (33). One limitation of our study lies in the fact that only one brand of FFP2 respirators has been tested for the preservation of its filtration properties through recycling treatment. Moreover, our experimental procedures deviated slightly from the standards NF EN 149+A1 (FFP2)/NF EN 14683+AC recommendations for assessing the filtrating performance and the inspiratory resistance of untreated and treated protective masks (35). Therefore, further investigations in line with the standard guidelines are required, in addition to ones using the actual SARS-CoV-2 instead of animal surrogate viruses. Another limitation lies in the approach of our demonstration that was carried out on the bench only: most surgical masks and FFP2 respirators tested herein had never been worn by nursing staff [noteworthy, a few pre-used masks were nonetheless moist heating treated and then tested; they exhibited neither difference in the SEM morphology nor in resistance at inspiratory flow (data not shown)]. In practice, further assessment in real life seems necessary to specifically address the saturation of filters with saliva/expectoration. Moreover, an evaluation of the impact of multiple treatment cycles seems required. However, other previous assays showed sustainment of all the face mask properties at the sealing surface up to 10 iterative treatment cycles based on moist-air heating (36), and our preliminary data herein did not show any negative impact for three to five cycles on structural integrity, altogether supporting the rationale for our approach.

## Conclusions

### Major Finding

Overall, our findings may pave the way in healthcare facilities for the reutilization of decontaminated, intact protective masks. Our study supports this straightforward strategy to circumvent surgical masks and N95/FFP2 respirators mass shortage, especially during the current COVID-19 pandemic.

### Perspectives

Should our recycling process be extended to other brands of surgical masks and N95/FFP2 respirators and thus be validated in the future by (inter-)national and sanitary authorities, mask decontamination could be recommended in hospital laundries and laboratories equipped with the adequate heat equipment.

## Data Availability Statement

The raw data supporting the conclusions of this article will be made available by the authors, without undue reservation.

## Author Contributions

All authors have seen and approved the manuscript. They all contributed significantly to the work. LB, GD, LV, AG, PL, PR, MS-T, and NH-V conceived the ideas. EB-M, JPa, LA, DB, SEy, P-IR, VV, JPo, PV, LM, SEs, MF, NE, YB, LB, PB, PR, and NH-V collected the data. SEs, JPo, NE, NH-V, MS-T, GD, J-PA, and PC contributed to the organization of the tests. GD, LB, EB-M, MF, JPo, PR, MS-T, and NH-V led the writing. The manuscript has not been previously published nor being considered for publication elsewhere.

## Conflict of Interest

NH-V has previously had a research contract with Aerogen. The remaining authors declare that the research was conducted in the absence of any commercial or financial relationships that could be construed as a potential conflict of interest.
