# Supplementary figures and images for: Controlled Heat and Humidity-Based Treatment for the Reuse of Personal Protective Equipment: A Pragmatic Proof-of-Concept to Address the Mass Shortage of Surgical Masks and N95/FFP2 Respirators and to Prevent the SARS-CoV2 Transmission
Source: Front Med (Lausanne). 2020 Oct 20;7:584036. doi: 10.3389/fmed.2020.584036 (PMC7607499; doi:10.3389/fmed.2020.584036)

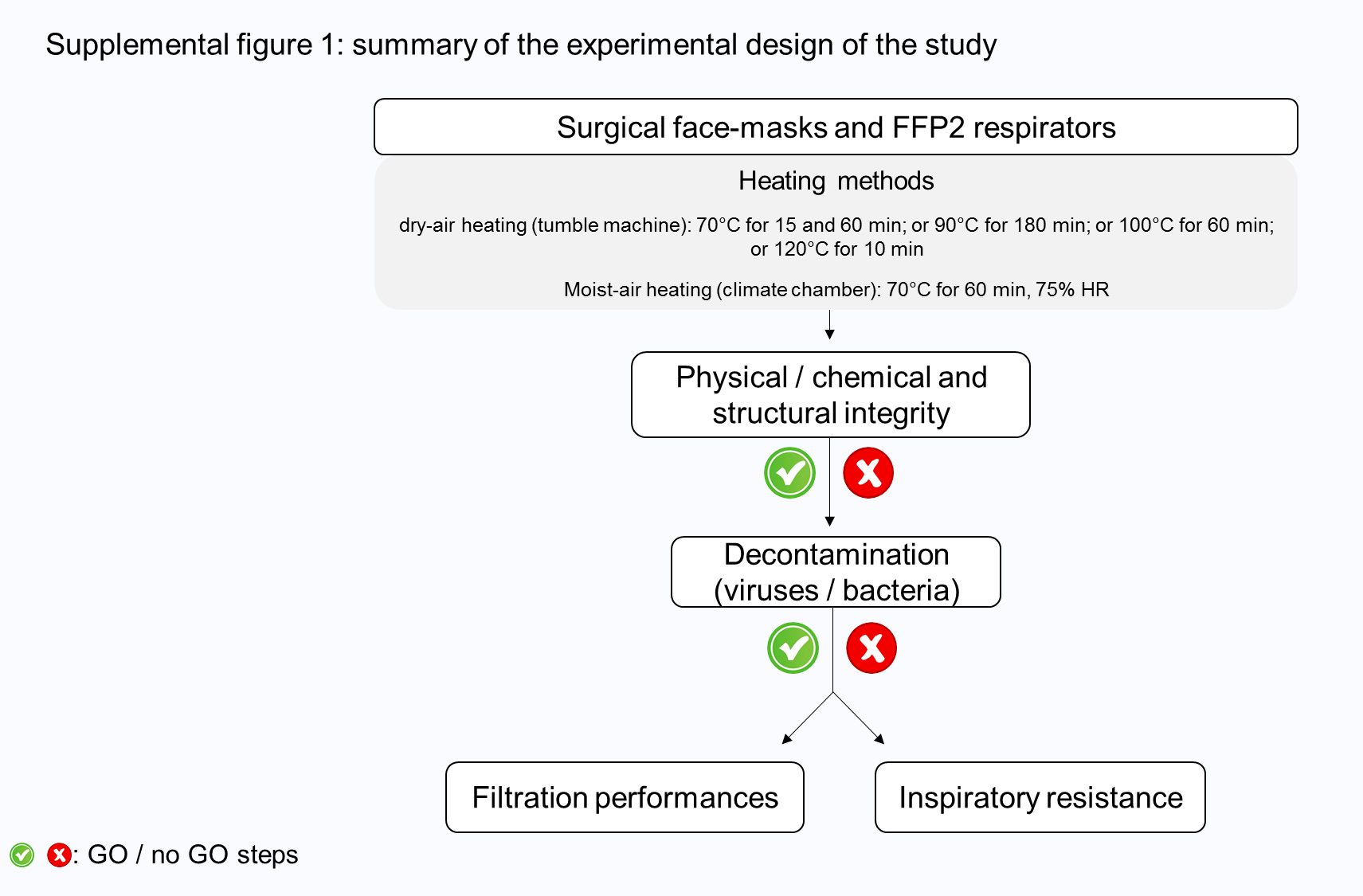

Supplement: Supplementary file 1 [file Image_1.tif]

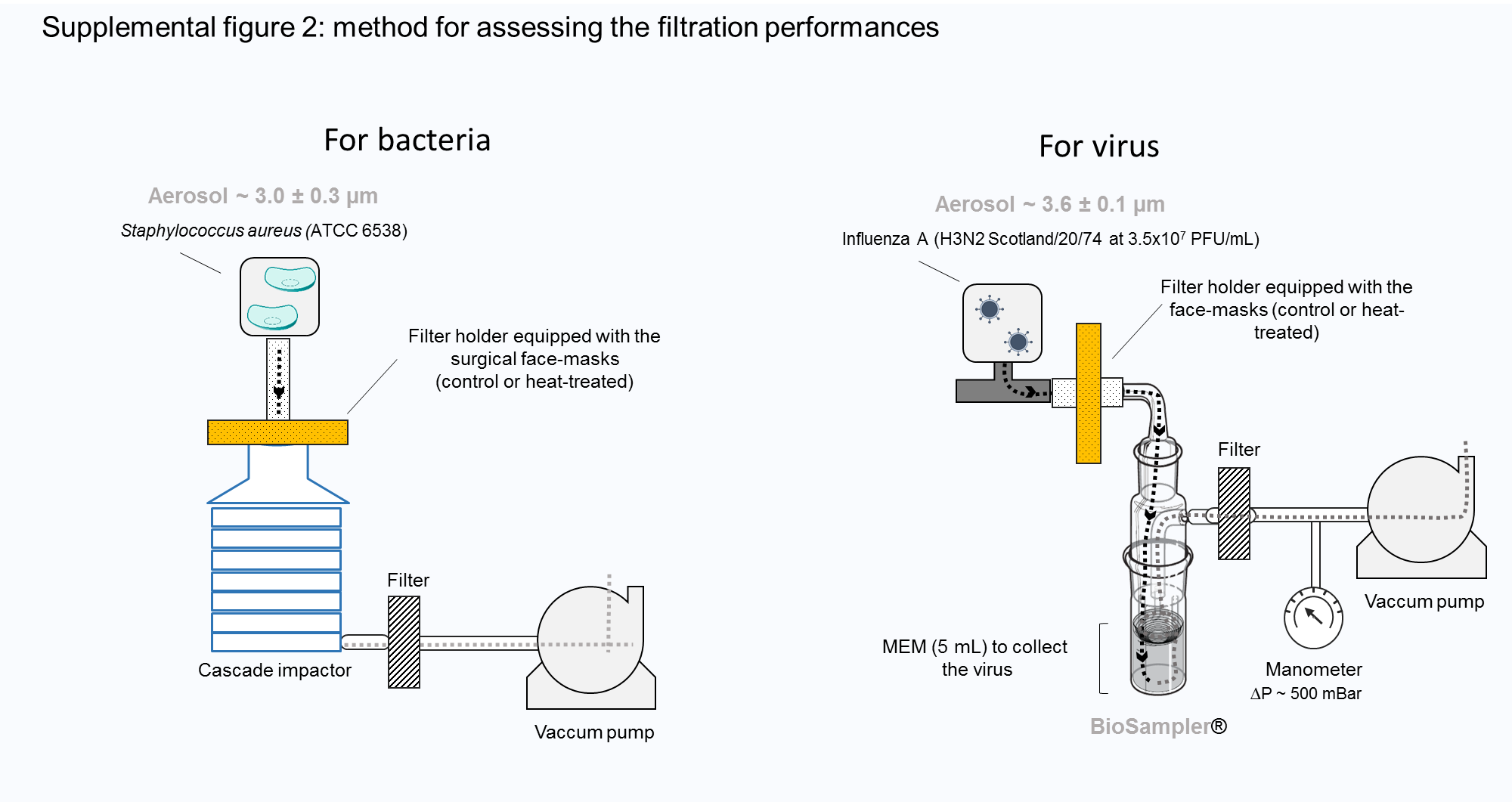

Supplement: Supplementary file 2 [file Image_2.tif]

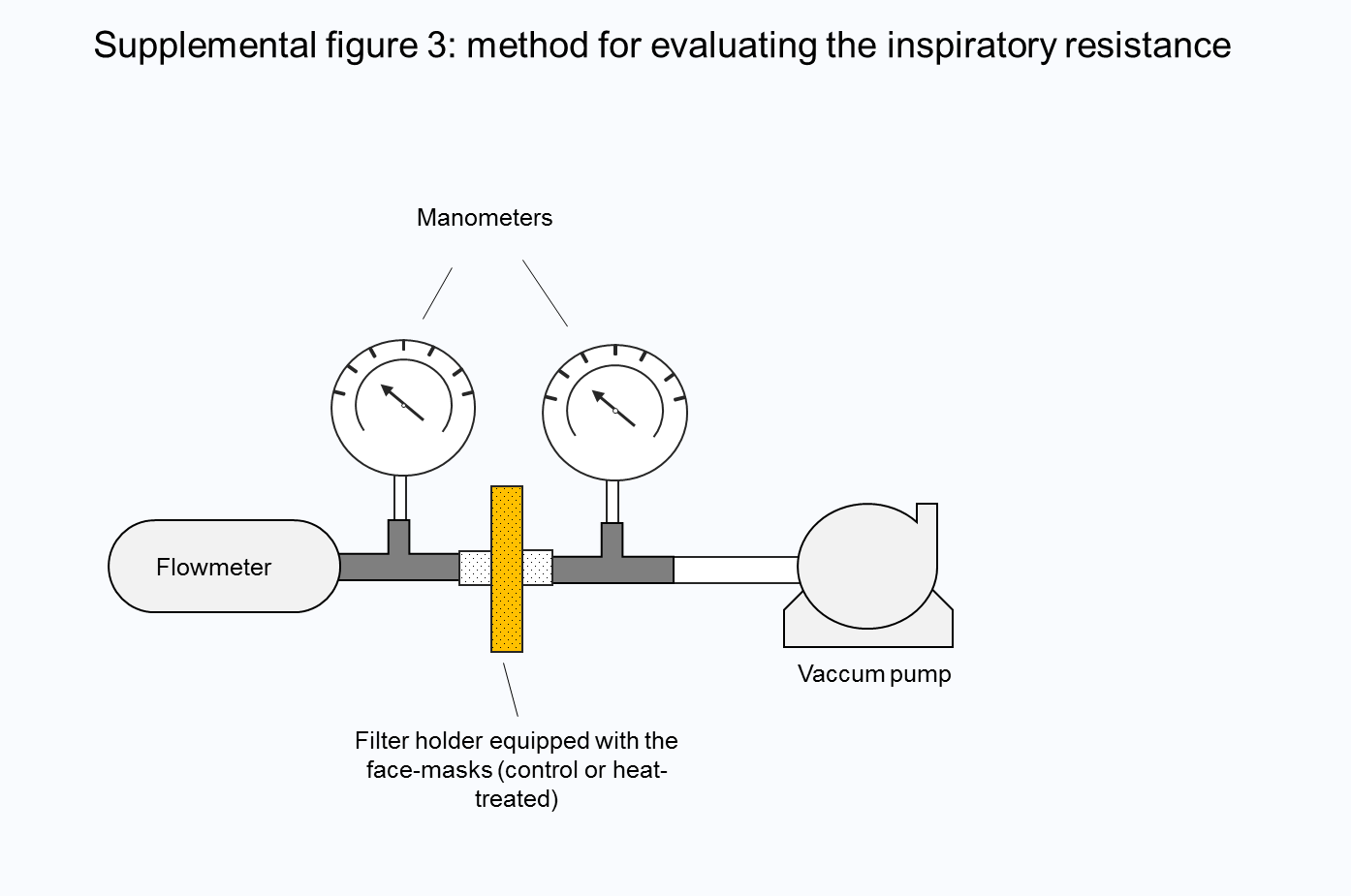

Supplement: Supplementary file 3 [file Image_3.tif]
